# A complex regional intervention to implement advance care planning in one town's nursing homes: Protocol of a controlled inter-regional study

**DOI:** 10.1186/1472-6963-11-14

**Published:** 2011-01-24

**Authors:** Jürgen in der Schmitten, Sonja Rothärmel, Christine Mellert, Stephan Rixen, Bernard J Hammes, Linda Briggs, Karl Wegscheider, Georg Marckmann

**Affiliations:** 1 Univ Dusseldorf, Medical Faculty, Department of General Practice, D-40225 Dusseldorf, Germany; 2 Institute for Bio-, Health and Medical Law, University of Augsburg, Universitätsstr. 2, D-86135 Augsburg, Germany; 3 Chair for Public Law, University of Bayreuth, Universitätsstr. 30, D-95447 Bayreuth, Germany; 4 Gunderson Lutheran Medical Foundation, 1836 South Avenue, La Crosse, WI 54601, USA; 5 Institute for Medical Biometry and Epidemiology, University Hospital Hamburg-Eppendof, Martinistr. 52, D-20246 Hamburg, Germany; 6 Institute for Ethics, History and Philosophy of Medicine, University of Munich, Lessingstr. 2, D-80336 München, Germany

## Abstract

**Background:**

Advance Care Planning (ACP) is an emerging strategy to ensure that well-reflected, meaningful and clearly documented treatment preferences are available and respected when critical decisions about life-sustaining treatment need to be made for patients unable to consent. In Germany, recent legislation confirms that advance directives (AD) have to be followed if they apply to the medical situation, but implementation of ACP has not yet been described.

**Methods/Design:**

In a longitudinal controlled study, we compare 1 intervention region (4 nursing homes [n/hs], altogether 421 residents) with 2 control regions (10 n/hs, altogether 985 residents). Inclusion went from 01.02.09 to 30.06.09, observation lasted until 30.06.10. Primary endpoint is the prevalence of ADs at follow-up, 17 (12) months after the first (last) possible inclusion. Secondary endpoints compare relevance and validity of ADs, process quality, the rate of life-sustaining interventions and, in deceased residents, location of death and intensity of treatment before death. The regional multifaceted intervention on the basis of the US program Respecting Choices^® ^comprises training of n/h staff as facilitators, training of General Practitioners, education of hospital and ambulance staff, and development of eligible tools, including Physician Orders for Life-Sustaining Treatment in case of Emergency (POLST-E).

*Participation data: *Of 1406 residents reported to live in the 14 n/hs plus an estimated turnover of 176 residents until the last possible inclusion date, 645 (41%) were willing to participate. Response rates were 38% in the intervention region and 42% in the control region. Non-responder analysis shows an equal distribution of sex and age but a bias towards dependency on nursing care in the responder group. *Outcome analysis *of this study will become available in the course of 2011.

**Discussion:**

Implementing an ACP program for the n/hs and related health care providers of a region requires a complex community intervention with the effect of nothing less than a cultural shift in this health care sector. This study is to our knowledge the first to develop a strategy for regional implementation of ACP, and to evaluate its feasibility in a controlled design.

**Trial Registration:**

ISRCTN: ISRCTN99887420

## Background

Decisions about life-sustaining treatment in patients unable to consent often pose difficult medical, legal and ethical questions, and may implicate emotional burdens for all actors involved, namely the patient concerned, the family, and the caregivers [1]. Typically, decisions to prolong life become more difficult as the burdens and risks of questionable treatments rise while their chances diminish. At the same time, institutions like nursing homes are bound to follow a decisional default directive to prolong life unless there is evidence that the individual would not consent or a medical order to the contrary [2].

Advance directives (ADs) have been advocated from the 1970ies as a means to limit life-sustaining treatment according to the preferences of the individual concerned [3]. Despite serious legal and policy efforts to propagate ADs in the population, notably the Patient Self Determination Act in the USA (1991), their prevalence has not exceeded 10-20%, and existent ADs prove often to be either not available or to have little relevance for the clinical decisions to be made. Perhaps worse, if they happen to be available and relevant, their validity often is not clear. Numerous debates and interventions with the aim to improve the prevalence, relevance and validity of ADs failed to make a lasting difference. Consequently, since the 1990ies many authors have argued that advance directives do and will not work, however well meant they may be [4].

On the other hand, the decisional challenges that lead to the call for ADs have remained unchanged: Life-prolonging therapy in an individual unable to consent may lead to an unwanted outcome - a risk that to a great amount depends on the frailty or co-morbidity given before the intervention becomes necessary. And in patients permanently unable to consent, we don't even know whether the current state may not already be completely unacceptable for the individual in the first place, let alone taking another burden and risk to prolong it. Modern medicine provides measures to attempt prolongation of life, but neither the foresight to know the individual outcome, nor an instrument that tells whether a given individual who is unable to consent is ready to take the risk.

Advance care planning (ACP) is a relatively recent approach that re-introduces advance directives, but within a completely new framework that has proved to answer many if not all of the objections, reservations and unsatisfying experiences regarding ADs in the past [5,6]. ACP rests on two crucial premises:

### 1. ACP is a process

In order to make an informed choice about future medical treatment under hypothetical medical conditions, an individual needs to appreciate possible future conditions and options, to develop personal preferences, and to discuss them thoroughly with family members, friends, and professionals. In other words, ACP requires a communicative process in its own right, comparable to but often more complex than obtaining informed consent for an imminent procedure. Therefore, decisions about future care have to be facilitated by specifically qualified personnel, be it trained physicians or, more realistically with view to universal resource constraints, trained non-physician staff, usually referred to as "facilitators". The facilitation process takes on average 1,5 hours [7], and it takes typically more than one encounter. In any case, the responsible treating physician should regularly be part of the facilitation process by finally confirming the decision-making capacity of the patient (or proxy), reviewing the chosen decisions with the patient and/or proxy, and signing - thus validating - the forms. Advance directives, then, are no more and no less than a useful formal expression of this essential, qualified communication process.

### 2. ACP involves a systemic approach

In order for the thus documented choices to be respected by all potential partners and interfaces of a patient's care, both in the ambulatory and in the hospital setting, effective ACP requires a systemic intervention addressing all relevant levels of care, and subsequently a systemic evolution that comes close to a micro-cultural change in this regard, involving numerous actors and structures. Issues like filing, updating, immediate access, and transferral of ADs to other levels of care need to be standardised and understood - their interpretation, if necessary, and finally adherence in medical decisions has to be trained and regularly evaluated.

ACP can be introduced in hospitals, and in the community. While its use in hospitals has shown to be effective and highly appreciated by patients and families [7], there are reasons to encourage implementation of ACP in the community [8]: Decision making may be improved if the patient is not under acute stress, and has more time to consider and appreciate different medical situations and treatment options. Further, some family members may be easier available, and the patient often has a long-standing trustful relation with his or her GP. Probably both approaches should be rather seen as complementary than as competing ways of ACP. In any case, a hospital admission should be seen as an opportunity to review any pre-existing ACP, be it during the hospital stay or thereafter.

Several regional ACP programmes have been developed in the US. One of them, Respecting Choices^®^, has been repeatedly shown to be effectively implemented in one community [8,6], and has been successfully transferred to Australia, where its local adaptation Respecting Patient Choices^® ^has been studied [7] and implemented in many local units.

In Germany, ADs are as little prevalent as elsewhere, although they have long been formally recognised to be binding by high court rulings, and the physicians' federal board. However, the patient advance directive law, enacted on Sept. 1^st ^2009, has added a new momentum to ACP, explicitly emphasising the patient's right to limit future life-prolonging treatments by means of an AD [9]. At the same time, the law requires legally binding ADs (a) to have written form and (b) to relate to concrete clinical decisions. While professional counselling is not explicitly required, many commentators now argue that most people will not be able to - and should rather not - make legally binding advance treatment decisions with possibly dramatic consequences before discussing them with a qualified health care professional.

However, qualified counselling with the goal to support a communicative process of ACP has not yet been developed in Germany as a standard procedure. In addition, medical professionals like nurses, emergency teams or physicians have not yet defined how to consider ADs appropriately in their decision making process. With few exceptions limited to hospice or critical care settings [10], physician orders for life-sustaining treatment (POLST, [11]) have not yet been implemented into routine care. Thus, notwithstanding recent legal endorsement of ADs in Germany there are no regional structures in place suggesting that effective ACP can be initiated, completed, or followed.

In nursing homes (n/h), the need for ACP is particularly obvious: Residents are usually characterised by high age, advanced frailty and/or chronic multimorbidity, conditions associated with a higher incidence of critical events, worse prognosis, and corresponding critical decision making. Many n/h residents suffer at some stage from conditions that make them permanently unable to consent, predominantly dementia and severe strokes. Empirical evidence indicates that many - not all - n/h residents wish to participate in ACP, and many of these choose to limit life-sustaining treatment [12]. At the same time, n/hs are institutions where residents live together with professionals trained to save lives, so without ACP, the default action usually is to initiate life-sustaining treatment, and call the emergency team which again follows standardised procedures directed to save lives.

This feasability study aims

(1) to develop an ACP program adapted from Respecting Choices^®^, suitable for the German medical, legal and ethical (cultural) context,

(2) to implement ACP in one town's nursing homes, and to ensure recognition of resulting plans in all related caregiver settings of this model region,

(3) to assess whether ACP leads to a higher prevalence of advance directives in general (primary) and of meaningful and valid advance directives in particular, compared with a control region,

(4) to examine in both groups whether clinical treatment is in accordance with the preferences expressed in existing advance care plans in residents transferred to hospital, and in deceased residents, and

(5) to study satisfaction of residents and their families, and of caregivers with the ACP planning process

## Methods/Design

### Definition: advance directive (by proxy)

As a patient advance directive (AD), we recognise any written document retrievable in the n/h charts specifying the resident's orders, preferences, wishes or thoughts regarding future medical treatment under hypothetical conditions, signed by the resident him- or herself.

From the (patient) AD we differentiate what we call an "AD by proxy", i.e. a written preference or order regarding future medical treatment under hypothetical conditions, signed not by the resident but by the legal proxy on the basis of the resident's substituted judgment [13].

### Team, and input from Respecting Choices^®^

The development of our ACP program involved medical, nursing, ethical, and legal (both medico-legal and socio-legal) issues that are reflected in the multi-disciplinary composition of our research team.

In June 2008, five team members (JidS, SR, CM, KL and GM) travelled for a training week to La Crosse, Wisconsin (USA), and obtained under the supervision of BH and LB the qualification as both Respecting Choices^® ^facilitators and instructors. In February 2009, BH and LB travelled to the German intervention town and participated in the one-week facilitator training. From the US, they accompanied and supported the subsequent development of an independent German program, based on but not identical with Respecting Choices^® ^http://www.respectingchoices.org.

### Ethical Approval

Ethical Approval of this study was granted by the ethics committee of the medical faculty of Düsseldorf (# 3116, 16.11.08).

### Study type and setting

In a longitudinal controlled study, we compare all 4 nursing homes (n/h) of the intervention town with 5 n/hs each in two separate control towns (convenience sample). The towns are located in three distinct governmental districts, and the distance between the towns is some 40 kms so as to prevent contamination.

### Centre Initiation Phase

In all regions, we first contacted the appropriate district government officials. With their support, we informed the directors of n/hs and hospitals, and invited them to take part in the study. Participating n/hs then recruited residents by forwarding our invitation to them (or their proxies, if recommended so by n/h staff), accompanied by a letter of recommendation of the respective n/h head, and the consent sheet. The letter was sent twice if residents were not responding to the first. For participation, residents had to return the signed consent sheet to the n/h staff. There were no recruitment incentives.

### Inclusion and exclusion criteria

All n/h residents were considered eligible to participate in the study.

During the centre initiation phase, originally considered exclusion criteria (regarding life expectancy, language barrier and short-term stay) were found to be of little relevance and unpractical for application by n/h staff and therefore judged to be skipped in order to simplify the study recruitment procedure.

### Time frame and data collection

Inclusion and observation began when all n/hs were ready for resident recruitment, i.e. 01.02.09. Originally, inclusion was intended to last until 30.04.10, with observation lasting two months longer than last inclusion, i.e. until 30.06.10.

However, the training of facilitators in the intervention region lead to an unforeseen recruitment bias: When regular facilitation was taken up by July 2009, the facilitators addressed intentionally chosen residents (e.g., because of imminent end-of-life decisions), and after completed facilitation invited them (or their proxies) to participate in the study. Thus from July 2009 a selected subgroup of residents was approached for the study in the intervention region, and because of the preceding close personal encounter, a high majority of these residents consented. When this bias was recognised, inclusion was retrospectively terminated as of 30.06.09 (confer corresponding entry in ISRCTN registration).

There has been no interim data analysis until this study protocol was submitted.

The collected data include demographic, medical, psychological and social baseline characteristics of the participants, in particular the degree of physical and cognitive impairment, further the presence of any ACP documents before and after intervention at the end of follow-up, the delivery of defined index interventions, and the circumstances of and prior to death. If available, copies of any written ADs were obtained.

Baseline data were extracted from a standardised interview with the resident, if capable, else with the proxy. Other sources for the baseline information are a standardised interview with a nurse, and the respective chart. Baseline items are listed in table 1.

**Table 1:** Baseline Data

Data Source	Items
resident (or proxy, if resident incapable)	age, sex, marital state, former profession, nationality, date of moving in, religious denomination and belief

	any advance care plan (AD); *if given: *place where it is thought to be kept; *if not given: *personal interest in ACP

	close contacts (relation and number); number of regular visitors; visits per month

caregiver	Barthel-Index; degree of dementia (GDS); resuscitation status

	number of regular visitors; visits per month

file	degree of dependency on nursing care; morbidity; presence of permanent feeding tube

	advance care plan (AD); if given: place where it is kept, and retrieval of a copy for further analysis

	designated health care proxy (custodian or durable power of attorney)

All participants were regularly followed up with respect to the existence of any ACP-related documents. The n/hs reported on a regular basis whether residents had been transferred to hospital, or died. Both events triggered specific follow-up data collection (items cf. table 2), either in the hospital or in the n/h if the resident died at home. Hospital follow-up was only possible if the resident had been referred to the respective local (in-town) hospital; referrals to other hospitals could not be followed because of limited resources.

**Table 2:** Observational Data

Data source	Items
n/h file (all participants)	hospital stays; death; evidence of advance care plan/advance directive, if given: further details

hospital file (if given)	mode and cause of hospitalization

	length of stay in the hospital/in the intensive care unit

	index surgical or medical interventions, including CPR, endotracheal ventilation (days), feeding tube insertion, endoscopy and CT/MRI imaging

	evidence of advance care plan/AD: filed copy or note in discharge letter

file and last caregiver of deceased residents	place of dying

	CPR before death

	presence of an advance care plan (e.g., AD, POLST) before death

	caregiver's evaluation of the amount of suffering before death and of the appropriateness of palliative care

	advance directive or physician's note of therapy restriction

### Endpoints

**Primary endpoint **is the prevalence of written patient or proxy ADs.

**Secondary endpoints **relate to relevance and validity of the identified (proxy) ADs, to process and clinical outcome parameters, and to possible influential factors. Outcome parameters compare extent and frequency of selected interventions, and the concordance of factual treatment with documented patient preferences.

In more detail, the following endpoints are considered for comparative analysis:

1. Process quality

1.1 Prevalence of (proxy) ADs (= primary endpoint)

1.2 Clinical relevance of the given (proxy) ADs

Criteria for clinical relevance of (proxy) ADs are taken to be whether

1.2.1 a health-care proxy has been designated and has signed the AD document *(pertinent to patient ADs only!)*, so there is a potentially well-informed interpreter of the patient's preferences if necessary

1.2.2 for the hypothetical case that cardiac arrest occurs out of the current state of health, the decision about cardiopulmonary resuscitation (CPR) follows clearly (although not necessarily explicitly) from the (proxy) AD

1.2.3 statements relevant for emergencies (like whether CPR should be attempted or not) are presented in a concise *emergency document *with unequivocal, explicit orders for the case of acute life-threatening illness (like cardiac arrest), as known from the US POLST form

1.2.4 for the hypothetical case of future severely progressed dementia *(pertinent to patient ADs only!)*, the AD guides decisions in case of (a) cardiac arrest (CPR?), (b) dysphagia (long-term feeding tube?), and (c) life-threatening infection (antibiotics?)

1.3 Validity of the given (proxy) ADs

Criteria for validity are taken to be whether

1.3.1 the designated proxy and/or another family member or any witness has also signed the AD *(pertinent to patient ADs only!)*, indicating that the individual has allowed the presumably closest social contact to discuss and possibly challenge his or her preferences

1.3.2 the (proxy) AD has also been signed by a physician confirming that the signing individual (resident or proxy) was capable of doing so and has understood the implications of his or her choices

1.4 Easy access to (proxy) AD, defined by:

1.4.1 presence of well visible references to a given advance care plan in the paper or electronic file (e.g. specific coloured dot or specific note on back of file)

1.4.2 original or copy of advance directive and/or physician orders is accessible on the ward

1.5 Transfer of (proxy) ADs to the hospital

1.5.1 presence of a copy of the (proxy) AD in the hospital file

2. Outcome quality

2.1 care of all residents (in accordance with stated treatment preferences, if available), defined by:

2.1.1 rate of index-interventions for life-sustaining treatment per resident-month: (i) CPR, (ii) feeding tube insertions (PEG), (iii) endotracheal ventilation, (iv) renal dialysis, (v) pace maker insertions, (vi) endoscopy, (vii) CT/MRI scans

2.1.2 rate per resident-month of (i) all hospital admissions, (ii) all but surgical hospital admissions

2.1.3 rate per resident-month of (i) all hospital days, (ii) all but surgical hospital days

2.2 care before death (in accordance with stated treatment preferences, if available)

2.2.1 location of dying (n/h or hospital)

2.2.2 number of transferrals to hospital in the 30 (90, 180, 360) days before death

2.2.3 (i) in-hospital and (ii) ICU days in the 30 (90, 180, 360) days before death

2.2.4 rate of index-interventions for life-sustaining treatment in the 30 (90, 180, 360) days before death: (i) CPR, (ii) feeding tube insertions (PEG), (iii) endotracheal ventilation, (iv) renal dialysis, (v) pace maker insertions, (vi) endoscopy, (vii) CT/MRI scans

**3. Possible influential factors **(all endpoints with all factors)

3.1 Sex

3.2 Age

3.3 Nursing home

3.4 Level of dependency on nursing care (I to III, as defined by § 14, SGB XI = German law regulating the nursing care insurance)

3.5 Global dementia score

3.6 Type of AD, i.e. patient AD or AD by proxy (only endpoints 1.1, 1.2 and 1.3)

### Hypotheses

The null hypothesis implies that the rate of advance directives in the intervention region is not different from that in the control region. The alternative hypothesis is that they are different.

### Sample Size Calculation

The n/hs in the intervention and in each of the two control towns, respectively, correspond with some 500 residents per town, or 500 residents in the intervention and altogether 1000 residents in the two control regions. Given a turnover rate of up to 30% per year, we reckoned with additional 625 residents moving in during the recruitment period (about 200 per town).

Provided a participation rate of 50-60%, we expected 400 participants in each town. Considering a possible dropout rate (participants dying before approached for basic interview, and facilitation), we expected a sample size of 360 in the intervention and altogether 720 in the two control towns.

According to the literature, and data of an own unpublished survey, we assumed the prevalence of any advance care plans to be between 10 and 20%. To demonstrate this difference at a test level of 5% two-sided with a power of 90%, 266 evaluable participants per random group are required. We decided to include more participants since there is considerable uncertainty in the estimates; we want to be able to cope with an unknown rate of loss due to missing values and to allow exploratory subgroup analyses for hypothesis generation.

### Statistical Analysis

Analysis will provide a comprehensive depiction with the methods of descriptive statistics

(a) on patient level, structured by regions and n/hs

(b) aggregated on n/h level, structured by regions

Both primary and secondary endpoints will be compared between intervention and control group by means of models with random effects. For this purpose, the n/hs will be included into the model as random effects, the regions (towns) as fixed effects, and patient characteristics with significant impact on the respective endpoint also as fixed effects. As a measure for the intervention effect, we will use the adjusted contrast estimator of the difference between intervention and control group.

The employment of models with random effects is suggestive due to the assumption that different cultures of counselling will develop between the respective n/hs, while within single n/hs (relatively) homogenous decisions are to be expected. The correctness of this assumption will be controlled by the calculation and testing of measures for the strength of the cluster effect.

### Control group

In the control group of 10 nursing homes in two other towns, there was no intervention (care as usual), and there were no incentives for participation. Both staff and residents were informed that the study aims to improve the scientific understanding of n/h residents' medical courses and treatments in case of severe illness and death, and to develop plans to improve honouring their treatment preferences.

### Intervention (I): Time frame

The multi-faceted intervention started in February 2009 with a 5-day-course for facilitators, recruited from the n/hs' social services and nursing staff, and a 4-hr-course for GPs. Various parts of this complex intervention continued throughout the study until the end of observation.

### Intervention (II): facilitator and GP training

Core of the intervention was the training of up to five nurses or social workers from each of the four nursing homes of a middle-sized town to become ACP facilitators. The training began with a week of intensive teaching (5 × 4 hours), combining interactive lectures, small group working, and role play sessions. The trainees were encouraged to continue role-playing for another month within their respective n/h teams, and then to start facilitating ACP with residents, first in pairs in order to give each other feedback, and at times with supervisors from the study team.

12 four-hour facilitator plenary sessions until the end of the observation period, i.e. 30.06.10, allowed for the discussion of experiences, extension and consolidation of knowledge, skills and understanding, identification of needs and barriers, and development of a sustainable group structure.

The training aims to enable the candidates to facilitate discussions on ACP. The facilitators are trained to help residents or their proxies understand possible future medical conditions and the treatment choices to be made, further to support them developing, communicating, and documenting their personal preferences.

Emanating from the Respecting Choices^® ^materials and considering German regional needs and requirements, the study team and facilitator group developed together in a feedback-driven, multi-step process the necessary forms and structures, notably:

• the patient AD,

• a 'proxy AD', i.e. a document in which the proxy lays down his or her best knowledge or approximation of the chronically incapacitated individual's treatment preferences

• Physician Orders for Life-Sustaining Treatment in case of Emergency (POLST-E) as an integral part of the (proxy) AD,

• guides for facilitating the conversation, and discussing the forms with residents,

• policies for the interaction between facilitators and others (residents, family, GPs, house staff), and

• policies for enacting, filing, updating, transferring, and implementing completed directives in the n/h.

The specifically designed program forms may only be used and signed by trained facilitators, and by trained general practitioners (GPs).

GPs may be expected to be experts in obtaining informed consent for medical procedures in general, and counselling for ADs in particular. However, given quite diverse levels of medical (palliative care), legal, and ethical knowledge, and because ACP is not a familiar concept in Germany, they were also viewed to need specific training. An initial 4-hour training, including small group and role play modules, is followed in the first year by quarterly meetings of 2 hrs to discuss experiences and conflicts, and deepen understanding. GPs take over responsibility to attest (1) the decision-making capacity of the patient or proxy signing the form, and (2) that the individual concerned has fully understood and appreciated the possible implications of his or her documented choices. For this purpose, they are asked to join and conclude the facilitation process when the patient or proxy and the facilitator have drawn up a draft version of the AD. In order to function effectively, facilitators and GPs need to become micro-teams, and work together with mutual respect and trust.

### Intervention (III): systemic implementation

Besides the facilitator training, the regional multifaceted intervention comprises close cooperation with the district government, repeated educational on-site meetings with hospital and ambulance staff, and development of interface procedures such as standards of how to apply the Physician orders for Life-Sustaining Treatment in Case of Emergency (POLST-E) effectively under the respective institutional conditions.

### Intervention (IV): resource requirement and incentives for participation

The participating nursing homes of the intervention region are asked to invest significantly into this project: Firstly by exempting the chosen facilitator personnel from regular work for the sake of training (altogether some 70 hrs), secondly by allowing them to regularly exert the facilitation work. Given a n/h with 100 residents, a median "turnover" of 30 residents per year, and a median gross duration of the facilitation process of 1.5 hours (once the facilitators are experienced), we estimated (and thus informed the n/hs) that *in the long run*, ACP with all new residents will take 45 hrs per year plus 15 hrs for updating and adjusting existing facilitations, i.e. 60 person hrs per year or 5 hrs per month. During the first (qualification) year of implementation, the required time may be up to twice as high.

The intervention n/hs do not receive any financial compensation for this investment. However, the comprehensive facilitator training by experts from multiple disciplines is provided free of charge. In addition, experience from other ACP programs indicates that the implementation of ACP saves time that is otherwise spent on stressful debates with relatives, or ethical case discussions. Moreover, there is reason to expect that the emotional burden on nursing staff is relieved if the residents' individual treatment preferences are known and the staff can assume that either implementing or forgoing disputable treatment measures is in accordance with the patient's wishes. These changes may have effects both on staff satisfaction and on the staff's sick-leave rate. Finally, we assume that offering a free ACP program to all residents is an effective competitive factor on the large n/h market - especially when other regional n/hs are adapting the program at the same time. Altogether, these effects sum up to a significant yield of the invested resources that were presented to the n/h leads.

General practitioners are offered free training, and paid an expense allowance of about € 40 for their concluding part in each facilitation process, documented by copies of the respective POLST-E. This is analogue to position # 34 in the German physician fee schedule for private services (GOÄ) which covers the thorough discussion of life-changing illness. So far, ACP is not covered by the statutory health insurances, so GPs will have to charge this fee privately after the end of the scientific implementation period.

Emergency services and hospital staff are offered no other incentives than the prospect of a system where n/h residents' treatment preferences for future health hazards are known and well-documented, so there will be a sound basis for their end-of-life decision-making.

### Participation of project partners

Almost all professional partners initially addressed to participate unequivocally welcomed the project. In detail, these were

• the three governments of the respective districts whom we first addressed to with the request to support us contacting all other potential project partners,

• the 4 n/hs addressed in the intervention town and

• 9 of the altogether 10 n/hs addressed in the two control towns (one n/h in the control region refused to participate because of other priorities and could be replaced by means of a single more request)

• the respective local hospitals of the three towns, where in all cases administrative and clinical heads cooperated.

In the intervention town, both medical and staff heads of emergency services and the administrative and clinical heads of the local hospital approved of the study concept and consented to participate. The 4 n/hs made initially 16 facilitator trainees available for the intervention training and subsequent process of regular facilitating ACP with residents.

In order to identify the GPs caring for the residents in the intervention town's n/hs, we asked the 4 n/hs for a list of all GPs tending to at least one resident. By merging these lists, we obtained a list of GPs ordered by the number of n/h residents they cared for. We invited all 32 GPs caring for altogether at least 3 residents, and finally trained 20 GPs caring for altogether > 85% of the residents in all four n/hs.

### Participation of residents and non-responder analysis

At the beginning of the observation 1406 residents were reported to live in the 14 n/hs. We could not retrieve factual turnover, but were told to assume a median turnover of 30% per year, i.e. 2,5% per month or 176 additional residents during the 5-months-inclusion time. Of these 1582 residents (474 in the intervention region and 1108 in the control region), 645 (41%) were willing to participate. The response rate was 38% (180 residents) in the intervention region and 42% (465) in the control region. After the first month of inclusion 616 residents were recruited, i.e. more than 95% of all participants.

Of the 937 residents who did not participate, we received data of 759 (81%) residents for non-responder analysis. Of the participants, 28 died or moved out of the n/hs before data could be gathered so that the non-responder analysis compares 617 responders with 759 non-responders. Non-responder analysis shows an equal distribution of sex and age but a bias towards dependency on nursing care in the responder group (table 3). In Germany, dependency on nursing care is differentiated according to social law (SGB XI, § 15) into three categories: category I (at least 1,5 to less than 3 hrs of daily nursing care), category II (at least 3 to less than 5 hrs) and category III (at least five hours), the last being over-represented in the responder group.

**Table 3:** Non-responder Analysis

	Responders (n = 617)	Non-Responders (n = 759)	p < 0.05
Age (means)	82,5	83,1	no*

Sex: % female	73,7	75,2	no'

Care dependency: % high degree (III)	25,6	14,9	yes'

advance care plan: % present	13,0	15,0	no'

*t-test of independent sample; 'χ^2 ^test

Analysis of outcome data of this ongoing study will be available later in 2011.

## Discussion

Implementing an ACP program for the n/hs and related health care providers of a region requires a complex community intervention with the effect of nothing less than a cultural shift in this health care sector. While there are few powerful descriptive studies of exceptional regions that have adopted and fully implemented an ACP program over many years, and also ACP implementation studies in n/hs, this study is to our knowledge the first to develop a complex strategy for regional implementation of ACP from level zero, and to evaluate its feasibility in a controlled inter-regional design.

## List of abbreviations

ACP: advance care planning; AD: advance directive; CPR: cardiopulmonary resuscitation; GP: General Practitioner; n/h: nursing home; POLST: Physician Orders for Life-Sustaining Therapy; POLST-E: Physican Orders for Life-Sustaining Therapy in case of Emergency

## Competing interests

The authors declare that they have no competing interests.

## Authors' contributions

JidS, GM and StR conceived the first outline of this study. Together with BH and SR, a detailed study concept was worked out. BH and LB taught the Respecting Choices^® ^facilitator and instructor courses to the other authors (except StR); during this process, the team members further developed the study and especially the intervention concept. SR and StR contributed the legal framework of the study, KW the statistical concept. JidS and CM conducted the controlled study. JidS, SR, GM, BH and LB participated in the multifaceted regional intervention. JidS wrote the first draft of this manuscript, all authors revised it critically and gave final approval.

## Pre-publication history

The pre-publication history for this paper can be accessed here:

http://www.biomedcentral.com/1472-6963/11/14/prepub
